# Genomic, Proteomic, and Phenotypic Spectrum of Novel O-Sialoglycoprotein Endopeptidase Variant in Four Affected Individuals With Galloway-Mowat Syndrome

**DOI:** 10.3389/fgene.2022.806190

**Published:** 2022-06-23

**Authors:** Malak Ali Alghamdi, Hicham Benabdelkamel, Afshan Masood, Narjes Saheb Sharif-Askari, Mahmood Y. Hachim, Hamad Alsheikh, Muddathir H. Hamad, Mustafa A. Salih, Fahad A. Bashiri, Khalid Alhasan, Tarek Kashour, Pilar Guatibonza Moreno, Sabine Schröder, Vasiliki Karageorgou, Aida M. Bertoli-Avella, Hisham Alkhalidi, Dima Z. Jamjoom, Ibrahim A. Alorainy, Assim A. Alfadda, Rabih Halwani

**Affiliations:** ^1^ Pediatric Department, College of Medicine, King Saud University, Riyadh, Saudi Arabia; ^2^ Medical Genetics Division, Department of Pediatrics, King Saud University, Riyadh, Saudi Arabia; ^3^ Proteomics Resource Unit, Obesity Research Center, College of Medicine, King Saud University, Riyadh, Saudi Arabia; ^4^ Sharjah Institute for Medical Research, University of Sharjah, Dubai, United Arab Emirates; ^5^ College of Medicine, Mohammed Bin Rashid University of Medicine and Health Sciences, Dubai, United Arab Emirates; ^6^ Neurology Division, Department of Pediatrics, King Saud University, Riyadh, Saudi Arabia; ^7^ Nephology Division, Department of Pediatrics, King Saud University, Riyadh, Saudi Arabia; ^8^ Pediatric Kidney Transplant Division,Organ Transplant Center, King Faisal Specialist Hospital and Research Centre, Riyadh, Saudi Arabia; ^9^ Cardiology Department, College of Medicine, King Saud University, Riyadh, Saudi Arabia; ^10^ CENTOGENE GmbH, Rostock, Germany; ^11^ Pathology Department, College of Medicine, King Saud University, Riyadh, Saudi Arabia; ^12^ Radiology and Medical Imaging Department, College of Medicine, King Saud University, Riyadh, Saudi Arabia; ^13^ Department of Medicine, College of Medicine and King Saud Medical City, King Saud University, Riyadh, Saudi Arabia; ^14^ Strategic Center for Diabetes Research, College of Medicine, King Saud University, Riyadh, Saudi Arabia; ^15^ Department of Clinical Sciences, College of Medicine, Sharjah Institute for Medical Research (SIMR), University of Sharjah, Sharjah, United Arab Emirates

**Keywords:** Galloway-Mowat syndrome, steroid-resistant nephrotic syndrome, GAMOS, proteomic, KEOPS complex

## Abstract

Galloway-Mowat syndrome is a rare autosomal recessive disease characterized by a unique combination of renal and neurological manifestations, including early-onset steroid-resistant nephrotic syndrome, microcephaly, psychomotor delay, and gyral abnormalities of the brain. Most patients die during early childhood. Here, we identified a novel homozygous O-sialoglycoprotein endopeptidase (OSGEP) variant, NM_017807.3:c.973C>G (p.Arg325Gly), in four affected individuals in an extended consanguineous family from Saudi Arabia. We have described the detailed clinical characterization, brain imaging results, and muscle biopsy findings. The described phenotype varied from embryonic lethality to early pregnancy loss or death at the age of 9. Renal disease is often the cause of death. Protein modeling of this OSGEP variant confirmed its pathogenicity. In addition, proteomic analysis of the affected patients proposed a link between the KEOPS complex function and human pathology and suggested potential pathogenic mechanisms.

## Introduction

Galloway-Mowat syndrome (GAMOS) is a rare autosomal recessive renal–neurological disease characterized by early-onset steroid-resistant nephrotic syndrome, microcephaly, and brain anomalies (Galloway and Mowat, 1968; Cohen and Turner, 1994). It was first described in 1968 in two siblings with microcephaly, hiatal hernia, and kidney disease (Galloway and Mowat, 1968). In 2014, Colin et al. first identified that *WDR73* variants caused GAMOS in two unrelated families (Colin et al., 2014). During the last 5 years, several studies have described different variants in *WDR73* (Colin et al., 2014; Ben-Omran et al., 2015; Jinks et al., 2015; Vodopiutz et al., 2015; Rosti et al., 2016; Jiang et al., 2017), *NUP107* (Rosti et al., 2017), *WHAMM* (Mathiowetz et al., 2017), and *PRDM15* (Mann et al., 2021) as a causal agent for GAMOS.

Recently, Braun et al. (2017) identified novel causative variants in four genes encoding the four “kinase, endopeptidase, and other proteins of small size” (KEOPS) subunits: *OSGEP*, *TP53RK*, *TPRKB*, and *LAGE3*, in 37 individuals from 32 families with GAMOS (Braun et al., 2017). *OSGEP* encodes the tRNA N6-adenosine threonylcarbamoyltransferase protein (OSGEP)*,* a subunit of the highly conserved KEOPS complex. The KEOPS complex controls the universal chemical change of tRNAs essential for translational accuracy. It has been implicated in telomere-associated DNA damage response (DDR) signaling and exhibits intrinsic DNA binding ability (Braun et al., 2017). Braun et al. (2017) knocked down genes encoding KEOPS subunits in human podocytes, resulting in impaired cell proliferation, translational attenuation, endoplasmic reticulum stress, activation of DDR signaling, increased apoptosis, and defects in actin regulation, which are possible pathogenic features of GAMOS (Braun et al., 2017). Independently, another study by Edvardson et al. (2017) reported a familial case of GAMOS with a homozygous *OSGEP* variant.

Here, we report genetic and proteomic data and the clinical characterization of an extended family with four affected members with GAMOS.

## Clinical Report

**FIGURE 1:**
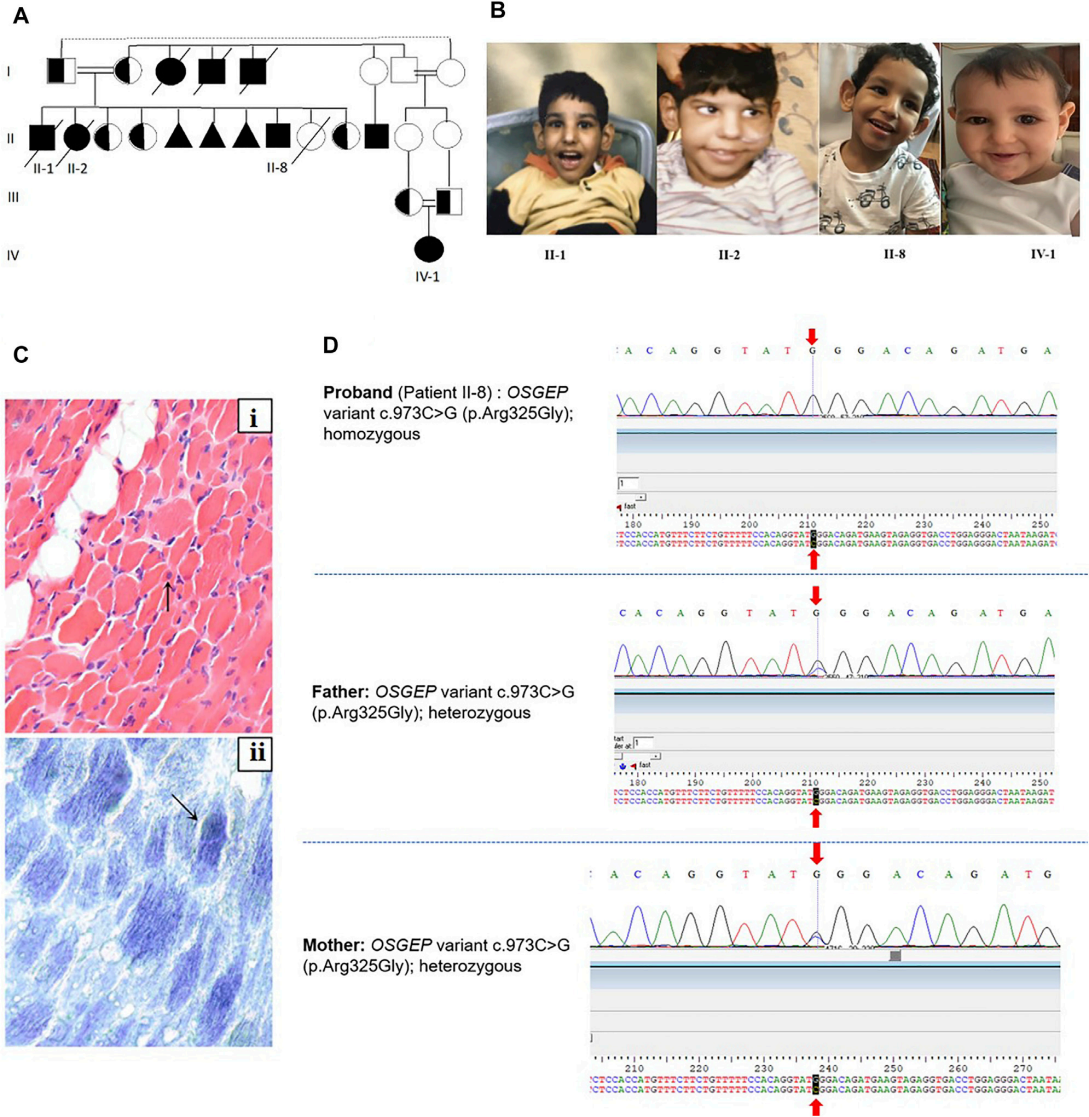
**(A)** Family pedigree. II-8 is the index patient. **(B)** Facial dysmorphic features of the three affected siblings. The facial dysmorphism includes microcephaly, almond-shaped eye, abnormally high but narrow forehead, ocular hypertelorism, depressed nasal bridge, and large and low-set ears. **(C)** Muscle biopsy of II-2: (i) Muscle biopsy shows a marked variation in fiber size with multiple scattered markedly atrophic fibers (arrow). Mild increased endomysial fibrosis and focal fat deposition are noted in the background (H&E, x400). (ii) Oxidative enzymes show mildly increased staining in scattered fibers (arrow, NADH, x400). **(D)** Sanger sequencing of the proband (II-8) and parents.

The parents of the patients were first cousins of Saudi origin. They have seven offsprings, three of whom exhibited strikingly similar phenotypes (II-1, II-2, and II-8) (Figure 1), and three spontaneous miscarriages. Table 1 summarizes the clinical features of the affected individuals.

**TABLE 1:** Clinical features of affected individuals.

Case	II-1	II-2	II-8	IV-1
Gender	Male	Female	Male	Female
Age of death	9 years	6 years	Still alive	Still alive
Ethnicity	Arabic	Arabic	Arabic	Arabic
Neonatal Profile				
Gestational age	Full-term	Full-term	Full-term	Full-term
Apgar at 5 min	N/A	N/A	9	9
Pregnancy course	Uneventful, SVD	Uneventful, SVD	Uneventful, c/s	Uneventful, SVD
Birth length	N/A	N/A	48 cm	48 cm
Birth weight	3000 g	3500 g	2700 g	3000 g
Birth head circumference	N/A	N/A	30.5 cm	34.5 cm
Renal phenotypes				
Onset of nephrotic syndrome	4 years	1.5 years	1.5 years	1 year (still asymptomatic, documented in the laboratory)
Renal biopsy	Mesangial proliferative glomerulonephritis	NP	NP	NA
Urinary tract abnormalities	(−)	(−)	(−)	(−)
Neurological features				
Brain MRI	Cerebral and cerebellar atrophy with extensive high signal intensity in the periventricular deep white matter	Moderate cerebrum and cerebellum atrophy and mild brainstem atrophy. Increased signal intensity in the periventricular white matter	No cerebral or cerebellar atrophy Mild high signal intensity in the periventricular and deep white matter with sparing of the subcortical white matter	Mild cerebral and cerebellar atrophy with extensive high signal intensity in the periventricular deep white matter
Cerebellar atrophy	(+)	(+)	(−)	(+)
Others	Facial dysmorphism	Facial dysmorphism	Facial dysmorphism	Facial dysmorphism

### Patient II-1

**FIGURE 2:**
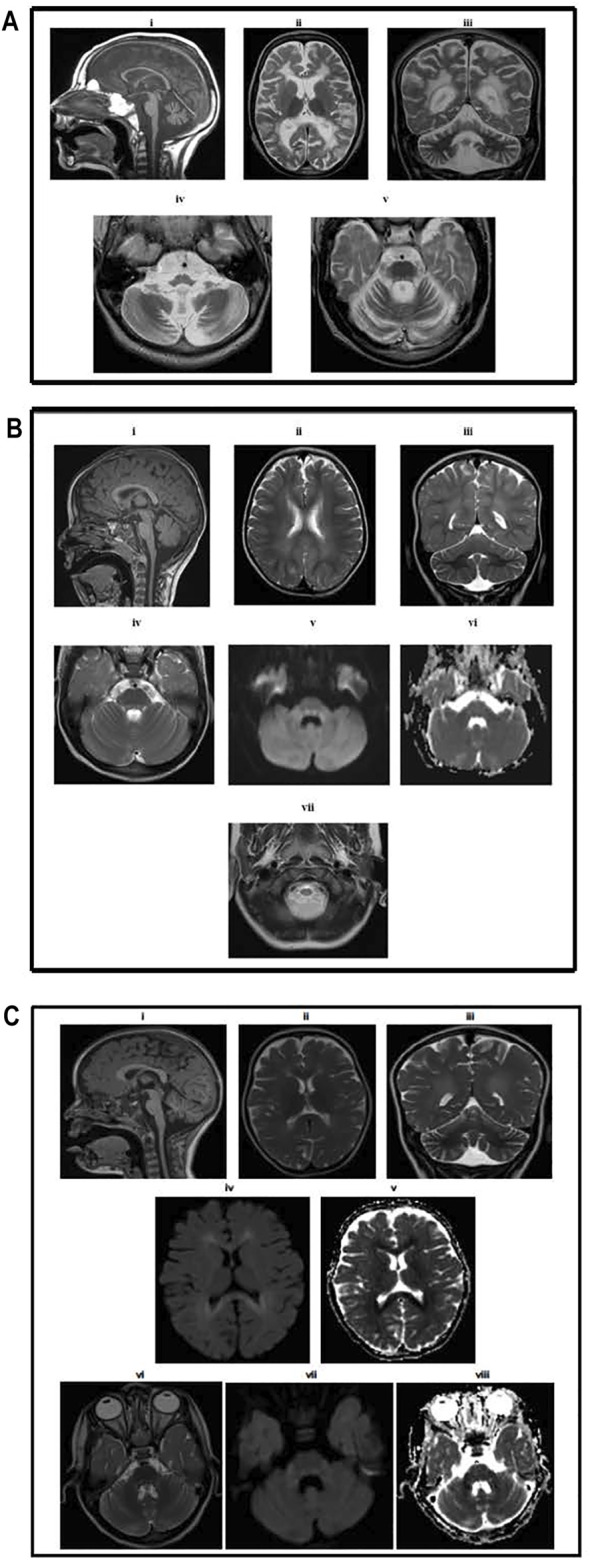
**(A)** Brain MRI results for Patient II-1. MR examination of the brain at the age of 5. (i) T1-weighted image showing atrophy of the corpus callosum and cerebellum and medulla oblongata and upper cervical spinal cord with normal-sized pons and midbrain. Axial (ii) and coronal (iii) T2-weighted images demonstrating extensive high signal intensity in the periventricular and deep white matter with relative sparing of the subcortical white matter. The cerebral and cerebellar atrophy is evident by the large ventricular system, sulci, and folia. Axial T2-weighted images at the level of the medulla oblongata (iv) showing medullary atrophy and at the level of pons (v) showing signal alteration, mainly involving the dorsal and peripheral parts of the pons. **(B)** Brain MRI result for Patient II-8. MR examination of the brain at the age of 2 years. (i) T1-weighted image showing the normal corpus callosum, cerebellum, and brainstem. The upper cervical spinal cord is atrophic. Axial (ii) and coronal (iii) T2-weighted images demonstrating mild high signal intensity in the periventricular and deep white matter, sparing the subcortical white matter. There is no cerebral or cerebellar atrophy. Axial T2-weighted (iv), diffusion-weighted (v) images, and the apparent diffusion coefficient map (vi) at the level of pons showing signal alteration mainly involving the dorsal and peripheral parts of the pons with diffusion restriction in the dorsal pons (vii). **(C)** Brain MRI results for patient IV-1. MR examination of the brain at the age of 18 months. (i) T1-weighted image showing mild atrophy of the cerebellum normal corpus callosum, brainstem, and upper cervical spinal cord. Axial (ii) and coronal (iii) T2-weighted images demonstrating mild high signal intensity in the periventricular and deep white matter, sparing the subcortical white matter. There is mild cerebral and cerebellar atrophy. Axial diffusion-weighted (iv) images and apparent diffusion coefficient map (v) at the level of the lateral ventricles showing diffusion restriction in the periventricular and deep white matter. Axial T2-weighted (vi), diffusion-weighted (vii), and apparent diffusion coefficient map (viii) showing signal alteration involving the dorsal pons with diffusion restriction.

The male baby was the first offspring of the family. No perinatal or neonatal problems were observed. The birth weight was 3.0 kg. He developed seizure and periorbital edema at 3 years of age and exhibited abnormal facial features (Figure 1). Considerable proteinuria was observed, and the patient was diagnosed with nephrotic syndrome and treated conservatively. MRI brain abnormalities are shown in Figure 2. He died at 9 years of age and had end-stage renal disease.

### Patient II-2

The female baby was the second offspring of the family. No perinatal or neonatal problems were observed. The birth weight was 3.5 kg. She developed the same symptoms as her brother, starting at 2.5 years of age, and died at the age of 6 with respiratory insufficiency, pulmonary edema, and cardiac tamponade.

This patient underwent two muscle biopsies for diagnostic purposes. The first was performed during her third year. It showed mild variation in fiber size and rare degenerating fibers. NADH and SDH showed preservation of intermyofibrillar sarcoplasmic architecture. Few fibers showed some degree of increased activity of the oxidative enzymes. COX stain was reported to be negative in the majority of the myofibers. The second muscle biopsy (Figure 1) was performed at the age of six. The variation in this biopsy was prominent, with multiple small-sized muscle fibers and a focal increase of the perimysial connective tissue in the form of increased fat deposition or mild fibrosis. However, the endomysial fibrosis was mild and focal. Like the previous biopsy, the intermyofibrillar architecture using NADH and SDH stains was relatively preserved with a focal increase of the activity of oxidative enzymes. Electron microscopy was performed on the second sample and showed a slightly increased number of mitochondria. No structural changes, nemaline bodies, or intramitochondrial inclusions were documented. Both samples lacked evidence of inflammation or structural abnormalities. Trichrome staining did not reveal evidence of ragged red fibers. Fiber type grouping using ATPases showed a normal mosaic pattern of fiber types with no evidence of grouping or selective fiber type atrophy.

### Patient II-8

This male patient was born at 37 weeks of gestation *via* emergency Cesarean section (C/S) due to previous C-section deliveries, with a birth weight of 2.7 kg, length of 48 cm, and head circumference (HC) of 30.5 cm (<3rd percentile). The Apgar scores at 1 and 5 min were 8 and 9, respectively. Soon after birth, the baby was admitted to the neonatal intensive care unit for hypoglycemia and respiratory distress, which was managed by Survanta^®^ *via* an endotracheal tube. He was then extubated and treated with a continuous positive airway pressure machine. After significant improvement, the patient was discharged in good health after 7 days. His mother noticed some dysmorphic facial features similar to those of his deceased siblings (Figure 1).

Initially, the patient did not have any symptoms, but the mother noticed delayed development. He started cruising at 1.5 years and walked with support at 1.8 years. He exhibited a delay in expressive language and communicated by pointing or facial/body gestures. He could say “mama” and “baba,” using the words appropriately for the parents but could not speak more words. He was able to follow simple one-step commands. At 4 years of age, he developed generalized spasticity with impaired mobility. There were no seizures, abnormal movements, or changes in consciousness or behavior. He was able to self-feed.

Physical examination revealed that apart from the previously mentioned dysmorphic features, generalized spasticity occurred all over the body, more in the lower limbs. Along with central hypotonia, the lower limb showed fixed flexion at the knee and ankle joints and flattening of the foot arches. Brisk deep tendon reflexes were also observed. At this point, different specialties, including pediatrics, neurology, ophthalmology, genetics, and metabolism were involved in caring for this patient for further assessment and evaluation. Laboratory investigations revealed hypoalbuminemia and hypomagnesemia with normal calcium levels, normal total creatine kinase, thyroid panel, ammonia, lactic acid, and metabolic screening. Urinalysis showed 3 + proteinuria, no hematuria, normal cell counts in urine, and normal renal function. EEG was normal. The 46XY karyotype was normal. The renal ultrasonography findings were unremarkable.

Figure 2 shows brain MRI, which revealed bilaterally symmetrical T2/flair hyperintensity and diffusion restriction in the periventricular white matter and dorsal brain stem with normal spectroscopy, suggestive of metachromatic leukodystrophy. These findings were similar to the synthesis defect of T6 modification of tRNAs, addressed by Edvardson et al. (2017) and Braun et al. (2017).

### Patient IV-1

She is the first baby of the young Saudi cousin-couple. She was an 18-month-old girl born at full term *via* normal spontaneous vaginal delivery (NSVD), with a birth weight of 3 kg, length of 48 cm, and HC of 34.5 cm. The Apgar scores at 1 and 5 min were 8 and 9, respectively. The patient was discharged in good health on the second day. She developed well, and her growth parameters at the age of 14 months were a weight of 11 kg (>75th centile), height 81 cm (>75th centile), and HC 44.5 cm (between the 10th and 25th centile). She received all the vaccines and follow-up care at a general pediatric clinic. Thereafter, her family started noticing some facial dysmorphic features similar to those of the affected cousins (Figure 1). However, she was asymptomatic at this age. Routine laboratory testing revealed proteinuria in urinalysis (++) (100–200), low albumin at 30 g/L, with normal prealbumin of 0.3 g/L, CBC differential, liver function test, electrolytes, blood gas, and renal profile. An MRI demonstrated similar imaging findings consisting of mild atrophy of the cerebellum normal corpus callosum, brainstem, and upper cervical spinal cord and also mild high signal intensity in the periventricular and deep white matter, sparing the subcortical white matter. There is mild cerebral and cerebellar atrophy (Figure 2C). Global parenchymal volume loss is seen in all patients.

## Materials and Methods

### Human Subjects

All patients underwent complete clinical evaluation of the GAMOS phenotype. Standard clinical exome consent was used for whole-exome sequencing of the whole family in the clinical laboratory. The parents signed an informed consent form. Ethical approval for clinical and laboratory data collection was obtained from King Saudi University (KSU) in Riyadh, Saudi Arabia, under protocol number Ref. No.18/0093/IR. Blood was then collected from the subjects and the parents in EDTA-coated tubes for DNA extraction, and the skin biopsy sample was taken from the index patient (II-8) and a healthy sister for proteomic profiling. Table 1 summarizes the clinical features of the affected individuals.

### Whole-Exome Sequencing

Whole-exome sequencing was performed for Patient II-8 as there were no DNA samples from the deceased siblings, patients II-1 and II-2. Approximately, 37 Mb (214,405 exons) of the consensus coding sequences were enriched from fragmented genomic DNA by >340,000 probes that were designed against the human genome (Nextera Rapid Capture Exome, Illumina, CA, United States). The generated library was sequenced on an Illumina NextSeq or platform (Illumina) to an average coverage depth of 70–100 ×. An end-to-end, in-house bioinformatics pipeline, including base calling, primary filtering of low-quality reads, probable artifacts, and annotation of variants, was applied.

All the disease-causing variants reported in HGMD^®^, ClinVar and all variants with a minor allele frequency of less than 1% in the ExAc database were considered for this study. Our evaluation focused on the exons with intron boundaries of ±20. All relevant inheritance patterns were considered, and the family history and clinical information were used to evaluate the identified variants. Only variants related to the phenotype have been reported.

### Computational Analysis of the Mutants

Protein modeling was performed to model the OSGEP amino acid variant exhibited by the patient. The crystal structure of the human probable tRNA N6-adenosine threonylcarbamoyltransferase (PDB accession number 6GWJ.1.C) was used as basis for modeling of the c.973C > G (p. Arg325Gly) variant using the Pymol program (pymol.org). Furthermore, *in silico* protein prediction algorithms (SIFT, PolyPhen, Mutation Taster) were used to predict the effect of selected mutation.

### Tissue Collection and Fibroblast Culture

A dermal biopsy pinch was collected from each patient (GAMOS and control) and transferred to the research laboratory in a medium containing advanced RPMI (Gibco, 12633012, MA, United States) and 1% antibiotic–antimycotic solution (Gibco, 15240-062). The tissues were left in the medium for 1–2 h after collection. Skin mesenchymal stem cells were then isolated separately using the explant method, as previously described (Alfares et al., 2020). In brief, fat was removed from the dermal biopsies, the tissues were chopped and plated in a six-well plate, and three pieces of tissue were collected in one well of a six-well plate. The cells were left undisturbed for 4–6 days in the conditioned medium (growth-specific medium) comprising advanced RPMI (Gibco, 12633012), 5% fetal bovine serum (FBS) (Gibco, 10099141), 20 pg recombinant human fibroblast growth factor (rh-FGF) (Lonza, CC-4065J), 0.1% insulin (Gibco, 51500056), and 1% antibiotic–antimycotic solution.

### Protein Extraction From Patient Cells

Proteins were extracted from triplicate biological samples of −2 × 10^6^ cells/mL from each patient (both GAMOS and control) directly in lysis buffer (0.5 ml, 30 mM Tris buffer pH 8.8 containing 7 M urea, 2 M thiourea, 4% Chaps, 1X protease inhibitor mix). The suspension was shaken for 1 h at room temperature and then sonicated using a microsonicator (Qsonica Sonicators, CT, United States) at 30% pulse and two intervals of 1 min each, separated by a minute’s gap on ice. Fifty millimolar dithiothreitol was then added, and the protein extracts were centrifuged at 20,000 × g for 1 h at 4°C. The pellets were then removed, and the solubilized proteins in the supernatants were precipitated using a 2D clean-up kit, according to the manufacturer’s protocol (GE Healthcare, IL, United States). The pellet was then solubilized in labeling buffer (7 M urea, 2 M thiourea, 30 mM Tris-HCl, 4% CHAPS, pH 8.5). The concentration of protein samples was determined in triplicate using a 2D-Quant Kit (GE Healthcare, IL, United States) (Galloway and Mowat, 1968).

### Fluorescence Labeling and Two-Dimensional Difference in Gel Electrophoresis

The extracted proteins (50 µg) from the patient’s sample and control were labeled with Cy3 and Cy5, respectively. Labeling was performed for 30 min on ice in the dark. The reactions were quenched by adding 1 µl of lysine (10 mM) for 10 min on ice in the dark. An equal amount of each sample was pooled, labeled with Cy2, and used as an internal standard. A dye-switching strategy was applied during labeling to avoid dye-specific bias (Supplementary Table S1). One-dimensional analytical gel electrophoresis was performed, followed by two-dimensional difference in gel electrophoresis on 12.5% fixed concentration gels, as previously described (Cohen and Turner, 1994; Alfadda et al., 2013). After two-dimensional difference in gel electrophoresis (2D-DIGE), the gels were scanned on a Typhoon 9410 scanner with Ettan DALT gel alignment guides using excitation/emission wavelengths specific for Cy2 (488/520 nm), Cy3 (532/580 nm), and Cy5 (633/670 nm).

### Protein Identification by Matrix-Assisted Laser Desorption Ionization–Time of Flight Mass Spectrometry

Coomassie-stained gel spots showing a significant difference between the groups were excised from the preparatory gel, washed, and digested according to previously described methods (Colin et al., 2014; Ben-Omran et al., 2015). Briefly, a mixture of tryptic peptides (1 μl) derived from each protein was spotted onto a matrix-assisted laser desorption ionization (MALDI) target (384 MTP Anchorchip, 800 μm Anchorchip, Bruker Daltonics, Bremen, Germany). As previously described (Alfadda et al., 2013; Benabdelkamel et al., 2015), MALDI-MS(/MS) spectra were obtained using an UltraflexTerm time-of-flight (TOF) mass spectrometer (MS) equipped with a LIFT-MS/MS device (Bruker Daltonics) at reflector and detector voltages of 21 and 17 kV, respectively, as described previously (Galloway and Mowat, 1968; Colin et al., 2014; Jinks et al., 2015; Vodopiutz et al., 2015). Peptide mass fingerprints (PMFs) were calibrated against a standard (peptide calibration standard II, Bruker Daltonics). The PMFs were assessed using Flex Analysis software (version 2.4, Bruker Daltonics). MS data were interpreted using BioTools v3.2 (Bruker Daltonics). The peptide masses were searched against the Mascot search algorithm (v2.0.04, updated on 09/05/2019, Matrix Science Ltd., United Kingdom). The identified proteins were screened for Mascot scores higher than 56 and *p* < 0.05.

### Bioinformatics Analysis: Pathway Analysis and Functional Classification of Proteins

The successfully identified proteins were uploaded into the ingenuity pathway analysis (IPA) software program (Ingenuity^®^ Systems, http://www.ingenuity.com). This program helps identify proteins and annotates them with related functions and pathways. The annotations were carried out by overlaying the proteins with their most significant networks and biochemical pathways from previous publications. The identified proteins were additionally classified into different categories according to their function and location using the protein analysis through the evolutionary relationship (PANTHER) classification system (http://www.pantherdb.org).

### Statistical Analysis

The 2D-DIGE gel images were uploaded into Progenesis SameSpots software (Nonlinear Dynamics, United Kingdom) and analyzed using an automated spot detection method. The analysis included comparing the samples from the GAMOS and control groups. Although an automatic analysis was performed to detect all the spots across all three gels, each selected spot was verified and manually edited wherever necessary. Normalized volumes were used to identify the differentially expressed spots. A cutoff ratio of ≥1.5-fold was considered significant. Student’s t-test was used to calculate statistically significant differences between the groups (*p* < 0.05 considered statistically significant). Spots that fulfilled the previously mentioned statistical criteria were subjected to further MS analysis. Principal component analysis (PCA) of the log-transformed spot data was performed.

## Results

### Whole-Exome Sequencing

The *OSGEP* variant NM_017807.3:c.973C>G (p.Arg325Gly) causing an amino acid change from Arg to Gly at position 325 was detected in the homozygous state in Patient II-8. This variant was detected in the parents in the heterozygous state. Variant segregation also showed that all healthy siblings were heterozygotes for the variant. Furthermore, other known monogenic causes of nephrotic syndrome or Galloway-Mowat syndrome have been excluded in the exome analysis.

### Computational Analysis of the Mutants

The amino acid R325 of OSGEP is evolutionarily highly conserved across species (Supplementary Figure S1), suggesting critical functional implications for protein structure. OSGEP protein modeling was based on human probable tRNA N6-adenosine threonylcarbamoyltransferase (PDB accession number 6GWJ.1.C) crystal structure identity (Supplementary Figure S1). The amino acid changed from large-sized arginine (hydrophilic, positively charged) to small-sized glycine (hydrophobic, neutral) which is close by the main catalytic active sides of OSGEP and was predicted to affect its interaction with tRNA (Braun et al., 2018). The main catalytic active sides of OSGEP are present in the following residues: H109, H113, Y130, N266, G177, E181, N266, and D294 (The UniProt Consortium, 2021). The previously reported disease-causing variant, R325Q, demonstrates a change from arginine (hydrophilic, positively charged) to glutamine (hydrophilic, neutral) at the same amino acid position. These results strongly suggest the pathogenicity of the c.973C >G (p.Arg325Gly) variant in the *OSGEP* gene. Furthermore, based on the human genome 37 reference, this variant is presumed to be deleterious and “probably damaging,” based on *in silico* protein prediction tools including SIFT and Mutation Taster. Using PolyPhen, the mutation predicted to be probably damaging with the score of 1.000, as well as Align-GVGD, revealed that this variant will most likely interfere with the function. Furthermore, MutationAssessor to assess the evolutionary conservation of the affected amino acid in protein homologs revealed a high score for this variant which suggests that it is likely to be deleterious. The mutation is present in the population gnomAD with an allele frequency of 0.0000040.

### Fluorescence Labeling and Two-Dimensional Difference in Gel Electrophoresis

**FIGURE 3:**
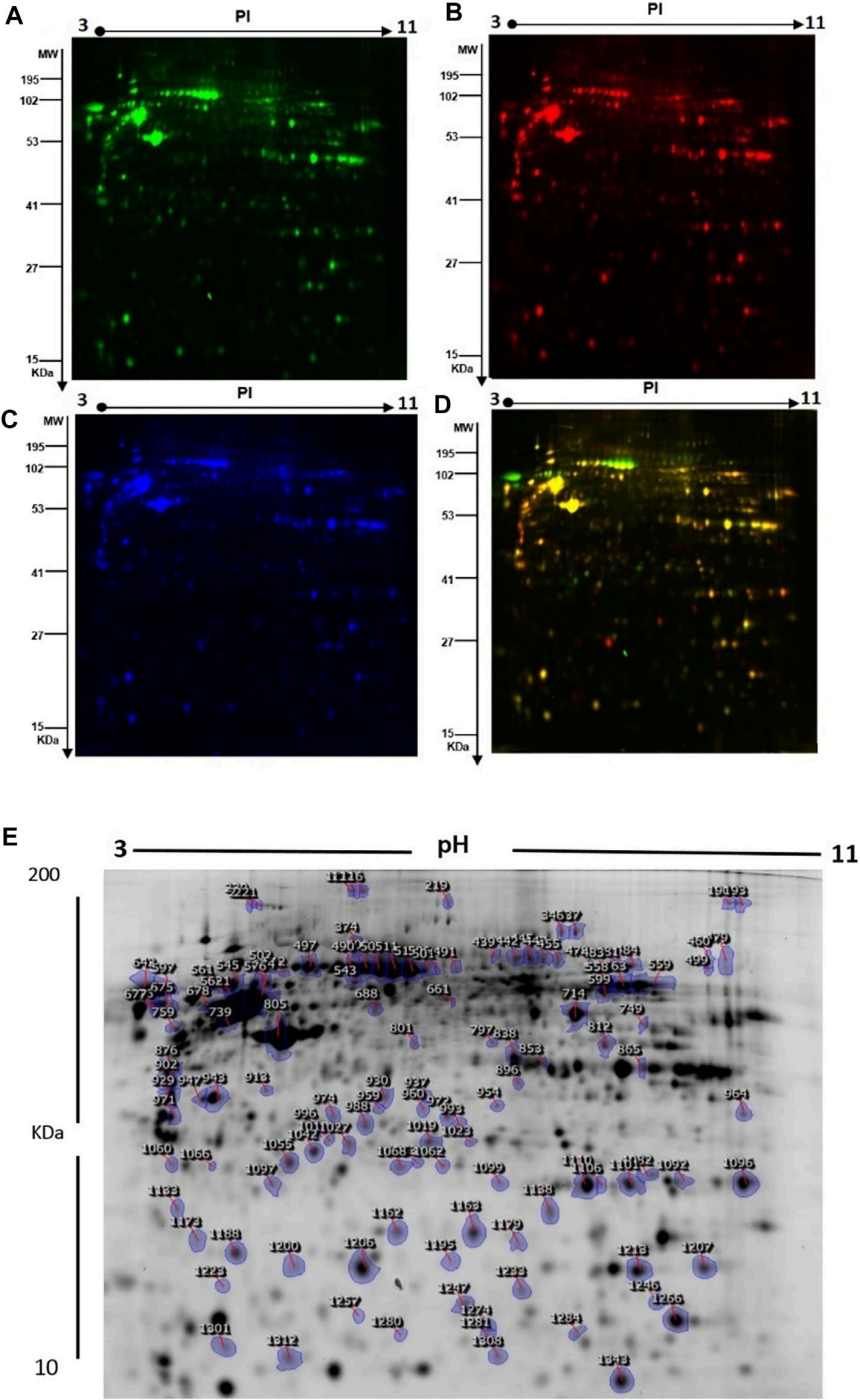
**(A)** Representative fluorescent protein profiles of the 2D-DIGE containing Cy3-labeled control samples. **(B)** Cy5-labeled samples from patients with GAMOS. **(C)** Pooled Cy2-labeled internal control. **(D)** Cy3/Cy5-merged 2D-DIGE comparison. **(E)** Representative image of protein spots from the skin fibroblasts. Numbered spots indicate those identified to be differentially expressed (over 1.5-fold change, *p* < 0.05) and successfully identified with MALDI-TOF/TOF.

The differences in protein levels between the patients with GAMOS (*n* = 3) and controls (*n* = 3) were assessed by 2D-DIGE. Representative fluorescent protein profiles of 2D-DIGE containing Cy3-labeled control and Cy5-labeled GAMOS samples, Cy2-labeled pooled internal control, and a merged Cy3/Cy5 comparison are shown in Figure 3A–D. A total of 1,450 spots were identified on the gels, of which 150 were significantly different (ANOVA, *p* ≤ 0.05; fold-change ≥1.5) between the GAMOS and control groups (Figure 3E). The spot patterns were reproducible across all three gels, leading to alignment and further analysis. Cy2-labeling (the internal standard) was included to permit normalization across the complete set of gels and quantitative differential analysis of the protein levels. The 150 spots showing significant difference between the two groups were then manually excised from the preparative gel for protein identification by MS.

### Protein Identification by Mass Spectrometry

To further characterize the proteins differentially expressed between patients with GAMOS and controls, PMFs were used. We successfully identified 107 of the 150 protein spots excised from the preparative gel. MALDI-TOF MS identified 97 spots as unique protein sequences and were matched to the SWISS-PROT database entries by Mascot with high confidence scores (Table 2, Supplementary Table S2). The sequence coverage of the proteins identified by PMF ranged from 8 to 87%. In a few cases, variants of the same protein were found at several locations on the gel (Table 2, Supplementary Table S2, Figure 3E). Among the 107 proteins identified, 40 protein spots were upregulated and 67 were downregulated in the samples from patients with GAMOS compared to those in the control group (Table 2, Supplementary Table S2, Figure 3E). Among the significantly upregulated proteins were RNA-binding protein 6 (up 4.2-fold, *p* = 7.36E-05), actin, cytoplasmic-1 (up 4.17-fold, *p* = 0.004), tropomyosin alpha-4 chain (up 3.26-fold, *p* = 6.05E-04), C18orf34 protein (up 3.10-fold, *p* = 0.006), macrophage receptor MARCO (up 3.10-fold, *p* = 0.01), peroxiredoxin-1 (up 3.06-fold, *p* = 0.004), and the RNA polymerase II transcription subunit 9 (up 3.0-fold, *p* = 0.006). The significantly downregulated proteins included keratin, type I cytoskeletal 16 (down 5.70-fold, *p* = 2.86E-06), serpin B12 (down 5.36-fold, *p* = 9.02E-04), 4-trimethylaminobutyraldehyde dehydrogenase (down 4.08-fold, *p* = 7.97E-05), galectin-1 (down 3.98-fold, *p* = 5.00E-04), and TGFβ1-induced anti-apoptotic factor 1 (down 3.68-fold, *p* = 5.85E-05). A complete list of upregulated and downregulated proteins is presented in Table 2. Among the identified proteins, a few proteins, including hexokinase-3, annexin A2, glyceraldehyde-3-phosphate dehydrogenase, nuclear mitotic apparatus protein 1, zinc finger protein 74, and dynein heavy chain 3, were found in more than one spot on the gels, which could be attributed to posttranslational modifications, cleavage by enzymes, or the presence of different protein species.

**TABLE 2:** Identified proteins, with changes in abundance between the control and patients with GAMOS.

Spot no.	Protein name	*p*-value (ANOVA)	GAMOS/control ratio
1206	Keratin, type I cytoskeletal 16	2.86E-06	−5.7**↓**
960	TGFB1-induced anti-apoptotic factor 1	5.85E-05	−3.68**↓**
1019	RNA-binding protein 6	7.36E-05	4.2**↑**
1173	4-trimethylaminobutyraldehyde dehydrogenase	7.97E-05	−4.08**↓**
1163	Zinc finger protein 423	8.39E-05	−3.22**↓**
1138	Probable imidazolonepropionase	1.17E-04	−2.5**↓**
929	Egl nine homolog 1	3.55E-04	−1.86**↓**
193	Transcription initiation factor TFIID subunit 7	3.60E-04	−1.27**↓**
1312	Galectin-1	5.00E-04	−3.98**↓**
1097	Tropomyosin alpha-4 chain	6.05E-04	3.26**↑**
1200	Serpin B12	9.02E-04	−5.36**↓**
225	Endoplasmin	0.001	2.07**↑**
1233	Peptidyl-prolyl *cis–trans* isomerase A	0.002	−1.34**↓**
759	Baculoviral IAP repeat-containing protein 3	0.002	−1.61**↓**
512	Protein-tyrosine kinase 6	0.002	1.37**↑**
1042	Annexin A5	0.003	1.5**↑**
221	Src substrate cortactin	0.003	1.4**↑**
1188	Heterogenous nuclear ribonucleoprotein C-like 1	0.003	−1.09**↓**
497	OTU domain-containing protein 4	0.003	−1.21**↓**
972	Annexin A1	0.003	1.55**↑**
1179	Zinc finger MYM-type protein 1	0.003	−2.41
801	Actin, cytoplasmic 1	0.004	4.17**↑**
1082	Peroxiredoxin-1	0.004	3.06**↑**
838	Annexin A2	0.004	−1.19**↓**
1110	Putative annexin A2-like protein	0.004	−1.51**↓**
116	Lipase member I	0.004	−1.7**↓**
812	Glyceraldehyde-3-phosphate dehydrogenase	0.005	−1.31**↓**
675	Uncharacterized protein C1orf122	0.005	2.58**↑**
1101	Elongation factor 1-alpha 1	0.005	1.64**↑**
559	Nuclear mitotic apparatus protein 1	0.005	−1.29**↓**
749	Stress-associated endoplasmic reticulum protein 1	0.005	−1.29**↓**
478	Zinc finger protein 74	0.005	−1.26**↓**
1092	Cofilin-1	0.005	−2.32**↓**
947	Complement factor H–related protein 5	0.006	−1.95**↓**
677	Mediator of RNA polymerase II transcription subunit 9	0.006	3**↑**
1162	Pleckstrin homology domain-containing family A member 2	0.006	−1.39**↓**
913	40S ribosomal protein SA	0.006	−1.67**↓**
996	Integrator complex subunit 13	0.007	−1.32**↓**
1133	Calmodulin-1	0.007	−1.29**↓**
230	Ubiquitin carboxyl-terminal hydrolase 21	0.008	1.69**↑**
1207	Peptidyl-prolyl *cis–trans* isomerase B	0.008	−1.3**↓**
501	Transformation/transcription domain-associated protein	0.008	1.94**↑**
930	Zinc finger protein 28 homolog	0.009	−1.34**↓**
337	Poly (ADP-ribose) polymerase 1	0.009	−1.83**↓**
853	Annexin A2	0.009	−1.17**↓**
1266	Profilin-1	0.009	−1.18**↓**
1062	Spectrin beta chain, non-erythrocytic 4	0.009	-2.3**↓**
455	Syntaxin-binding protein 3	0.009	1.6**↑**
599	Dynein heavy chain 3, axonemal	0.01	−1.17**↓**
513	Macrophage receptor MARCO	0.01	3.1**↑**
122	Developmentally regulated GTP-binding protein 1	0.011	−1.6**↓**
1343	Beta-galactosidase-1–like protein 3	0.011	1.41**↑**
1099	Annexin A2	0.011	2.05**↑**
993	F-actin-capping protein subunit beta	0.013	−1.39**↓**
442	Zinc finger protein 74	0.013	1.84**↑**
483	Actin-related protein 2/3 complex subunit 2	0.015	−1.31
937	Protein maestro	0.016	−2.51
219	NADH dehydrogenase (ubiquinone) complex I, assembly factor 6	0.017	1.51**↑**
714	Pyruvate kinase PKM	0.017	−1.16**↓**
481	Piwi-like protein 4	0.018	−1.56**↓**
507	Nesprin-2	0.019	−2.3**↓**
563	Dynein heavy chain 3, axonemal	0.019	−1.45**↓**
374	Kelch repeat and BTB domain-containing protein 12	0.02	−1.78**↓**
1023	Polycomb protein SUZ12	0.021	1.45**↑**
500	Hexokinase-3	0.021	2.16**↑**
491	Interferon regulatory factor 8	0.021	1.45**↑**
971	Macrophage receptor MARCO	0.021	−1.33**↓**
1246	Nucleoside diphosphate kinase B	0.023	−2.6**↓**
346	Regulating synaptic membrane exocytosis protein 1	0.023	−1.8**↓**
445	Allergin-1	0.023	1.88**↑**
543	Exocyst complex component 1	0.026	−1.32**↓**
555	Zinc finger protein Aiolos	0.027	−1.38**↓**
439	Poly (ADP-ribose) polymerase 1	0.027	1.755**↑**
964	Heterogenous nuclear ribonucleoprotein A1	0.028	−3.21**↓**
490	Hexokinase-3	0.029	1.67**↑**
1055	Probable ubiquitin carboxyl-terminal hydrolase MINDY-4	0.031	2.42**↑**
661	Carbohydrate sulfotransferase 7	0.034	−2.26**↓**
896	Receptor of activated protein C kinase 1	0.037	−1.28**↓**
739	Tubulin beta chain	0.039	−1.79**↓**
1106	Transgelin	0.039	−1.64**↓**
1096	Actin, cytoplasmic 1	0.041	−1.64**↓**
805	Actin, cytoplasmic 2	0.041	−1.22**↓**
1213	Destrin	0.043	−1.22**↓**
545	ATP-dependent RNA helicase TDRD9	0.049	1.74**↑**
865	Glyceraldehyde-3-phosphate dehydrogenase	0.049	−1.31**↓**
232	Heat shock protein HSP 90-beta	0.051	1.26**↑**
410	Nuclear mitotic apparatus protein 1	0.054	−1.52**↓**
813	Protein Shroom2	0.054	−1.28**↓**
738	Calumenin	0.054	1.71**↑**
554	Vascular endothelial growth factor C	0.06	−1.27**↓**
1059	14-3-3 protein zeta/delta	0.06	1.29**↑**
576	Apoptosis-inducing factor 3	3.12E-04	1.6**↑**
1280	Zinc finger protein with KRAB and SCAN domains 5	5.87E-04	1.51**↑**
1281	Putative E3 ubiquitin-protein ligase UBR7	0.001	1.97**↑**
943	Tropomyosin beta chain	0.003	−1.8**↓**
506	T-complex protein 1 subunit theta	0.005	2.54**↑**
642	C18orf34 protein	0.006	3.1**↑**
499	Non-POU domain-containing octamer-binding protein	0.006	1.42**↑**
1027	Basic immunoglobulin–like variable motif-containing protein	0.008	−1.35**↓**
1308	MICOS complex subunit MIC60	0.009	−1.31**↓**
988	Uncharacterized protein C2orf50	0.015	−1.34**↓**
132	Plasma kallikrein	0.017	−1.73**↓**
511	Synaptonemal complex protein 2	0.017	2.75**↑**
974	Elongation factor 1-delta	0.022	−1.21**↓**
621	Protein OSCP1	0.025	1.76**↑**
1060	Tropomyosin alpha-1 chain	0.041	−1.46**↓**
447	Heat shock cognate 71 kDa protein	0.049	1.67**↑**

The table shows average ratio values for control and GAMOS samples, with their corresponding levels of fold changes and one-way ANOVA (*p*-value < 0.05), using 2D-DIGE (Analysis type: MALDI-TOF; database: SWISS-PROT; taxonomy: *Homo sapiens*, **↓** Downregulated, **↑** Upregulated).

### Principal Component Analysis and Cluster Analysis

PCA was performed using Progenesis SameSpots software to determine and visualize the potential clustering of the proteins that were differentially expressed in the patients with GAMOS compared to the control group. PCA was performed on all 107 spots that exhibited statistically significant changes (ANOVA, *p* < 0.05) in abundance as identified by MS. The analyses revealed that the two groups clustered distinctly based on different proteins with an 84.2% score (Supplementary Figure S2). The differentially abundant spots showed expression pattern clusters according to their abundant patterns based on a hierarchical clustering analysis (Supplementary Figure S3). The clustering pattern showed that the change in the protein intensities for the selected spots between the GAMOS and control samples was significantly different.

### Protein–Protein Interaction Mapping

**FIGURE 4:**
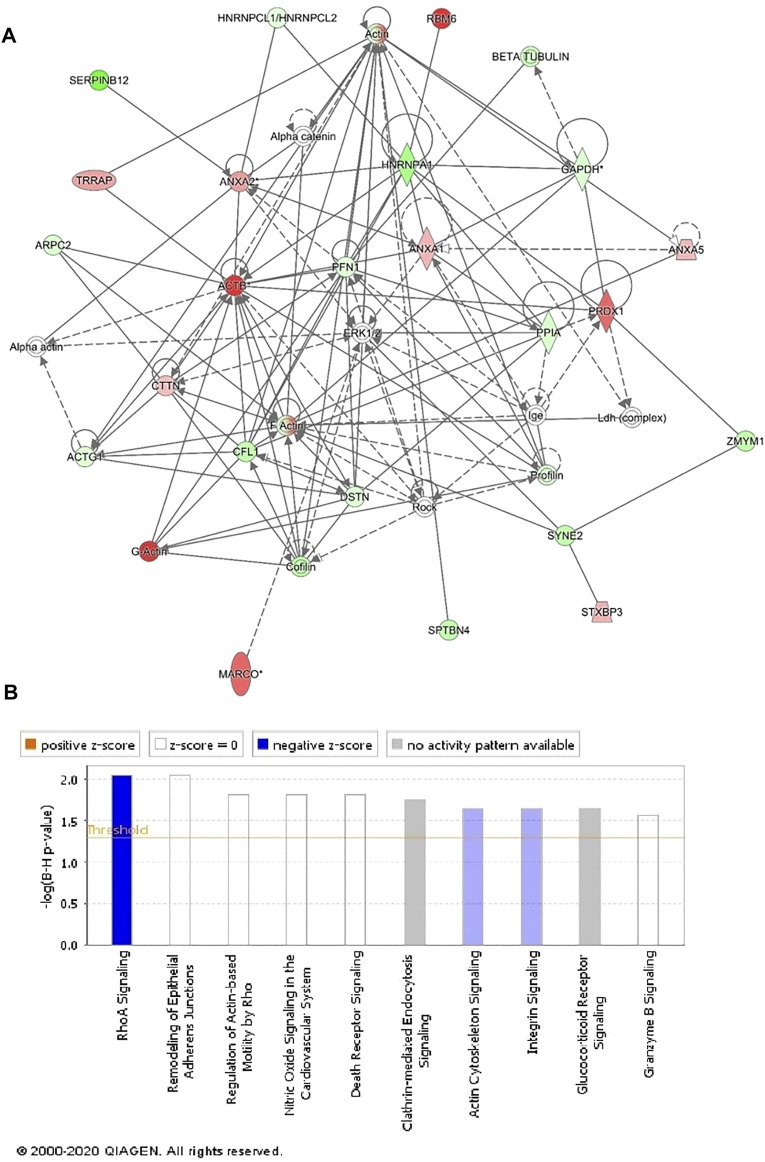
Schematic representation of the most significant IPA networks involving the differentially regulated proteins between the GAMOS and control samples. IPA analysis found that the functional interaction network pathway with the highest score was related to “cellular assembly and organization, cellular function and maintenance, and tissue development.” This pathway incorporated *PFN1*, *ERK1/2*, and *ACTB* as central nodes downregulated in patients with GAMOS. Nodes in green and red correspond to downregulated and upregulated proteins, respectively. **(A)** Colorless nodes were proposed by IPA and suggest potential targets functionally coordinated with the differentially abundant proteins. Solid lines indicate direct molecular interactions, and dashed lines represent indirect interactions. **(B)** Diagram shows the top canonical pathways ranked by the *p*-values obtained by the IPA.

We next performed protein–protein interaction analysis for the 107 differentially regulated proteins in patients with GAMOS using IPA. The analysis revealed that among 107 proteins, 33 proteins interacted either directly or indirectly *via* protein networks (Figure 4A). The software computes a score based on the best fit obtained from the input data set of proteins and the biological functions database to generate a protein–protein interaction network. The generated network is preferentially enriched for proteins with specific and extensive interactions. The interacting proteins are represented as nodes and their biological relationships as a line. Based on the data, three interaction networks were identified for proteins exhibiting differential expression profiles. The highest scoring network (score = 50; Figure 4, Supplementary Figure S4) incorporated 29 proteins. The proposed highest-interaction network pathway was related to cellular assembly and organization, function and maintenance, and tissue development. Only the top pathways are shown in Figure 4A. The canonical pathways enriched in the current dataset are shown in Figure 4B. These canonical pathways are sorted down to a decreasing log (*p*-value) of enrichment. The three most interesting enriched canonical pathways included RhoA signaling, actin cytoskeleton signaling, and integrin signaling.

### Functional Characterization of the Differentially Expressed Proteins

Following MS analysis, all 107 differentially abundant proteins identified between the GAMOS and control samples were subjected to the PANTHER classification system (http://www.pantherdb.org) for classification according to their function (Supplementary Figure S5A) and location (Supplementary Figure S5B). The dominant functional categories identified were binding proteins (49%) and proteins involved in catalytic activity (28%) (Supplementary Figure S5A). Most proteins showed cellular localization (44%), followed by organelle-localized proteins (26%) (Supplementary Figure S5B).

## Discussion

GAMOS is a phenotypically heterogenous disease characterized by a combination of early-onset steroid-resistant nephrotic syndrome and microcephaly with brain anomalies (Galloway and Mowat, 1968; Cohen and Turner, 1994; Braun et al., 2017; Braun et al., 2018). The three siblings (II-1, II-2, and II-8) and their cousin from a consanguineous family share a similar phenotype, including dysmorphic facial features, respiratory distress, and nephrotic syndrome, resulting in massive proteinuria and brain abnormalities. Their phenotypes were similar to those of patients with *OSGEP* variants reported by Braun et al. (2017), which included microcephaly, severe deformation of the forehead, reduced gyration, diffuse cortical atrophy, focal segmental glomerulosclerosis, and foot process effacement resulting from massive proteinuria (Braun et al., 2017).

The homozygous variant, c.973C>G (p.Arg325Gly), in the *OSGEP* gene identified in Patient II-8 by whole-exome sequencing caused an amino acid change from arginine to glycine, which was presumed to be deleterious and probably damaging based on *in silico* protein prediction algorithms. While this particular variant has not been described previously in the literature, two different variants that affect the same amino acid residue have been published as disease-causing for Galloway-Mowat syndrome (Braun et al., 2017; Edvardson et al., 2017). Therefore, the variant detected here is classified as “probably pathogenic”, according to the American College of Medical Genetics and Genomics (ACMG) recommendations. Retrospective clinical analysis revealed compatibility of the patient’s phenotype with the identified variant, and variant segregation showed that all healthy siblings were heterozygotes for the variant.

We identified multiple differentially dysregulated proteins, indicating that OSGEP plays a role in multiple developmental pathways. Thereby, these dysregulated pathways might be involved in GAMOS pathology. For example, the downregulated proteins with the highest GAMOS/control ratio regulate supramolecular fiber organization, cyclic-nucleotide phosphodiesterase activity, modification by a host of symbiont morphology or physiology, and smooth muscle contraction (Supplementary Figure S6). Proteins that showed the highest GAMOS/control ratio were involved in the P2X7 receptor signaling complex, vesicle-mediated transport, translocation of *SLC2A4* (GLUT4) to the plasma membrane, and phagocytosis (Supplementary Figure S7). The renal–neurological presentation of GAMOS could be traced to the protein dysregulation found in this study. P2X7 signaling activation, which includes upregulation of ACTB, HSPA8, and HSP90AB1, has been shown to play a significant role in brain development and has been linked to many neurodegenerative, neuroinflammatory, and neurogenic diseases (Sharp et al., 2008; Andrejew et al., 2020). Furthermore, heat shock proteins (HSPA8 and HSP90B1), annexins (ANXA1, ANXA2, and ANXA5), and vesicle-mediated transport proteins (ACTB, CTTN, STXBP3, MARCO, and YWHAZ), which are essential for normal kidney development, are found to be dysregulated and to contribute to the observed nephrotic syndrome (Smoyer et al., 1996; Markoff and Gerke, 2005; Simsek et al., 2008; Chebotareva et al., 2018). Interestingly, none of the deferentially regulated genes described is a member of the KEOPS complex nor is it a known GAMOS-causing or NS-causing gene. Indeed, the deferentially regulated genes that are suggested to be involved in kidney pathology are important during development but are not a known cause of monogenic nephrotic syndrome.

Taken together, the data have suggested that the loss of tRNA N6-adenosine threonylcarbamoyltransferase protein function affects many other essential proteins required for protein folding, protein binding, transport, and catalytic activity.

## Data Availability

The datasets for this article are not publicly available due to concerns regarding participant/patient anonymity. Requests to access the datasets should be directed to the corresponding author.

## Supplementary Material

Supplementary Figure S1 OSGEP protein 3D model. **(A)** Crystallized structure of the human probable tRNA N6-adenosine threonylcarbamoyltransferase (PDB accession number 6GWJ.1.C). **(B,C)** Protein structure is shown as teal ribbon, while the amino acids at the mutation site are displayed as red stick. The catalytic binding sites of OSGEP are mapped as blue spheres (Residues: H109, H113, Y130, N266, G177, E181, N266, and D294). **(D)** Surface electrostatic potential surrounding Arg325, with the protruding positive patch generated by the arginine, which is lost in the bottom–right picture **(E)** after the replacement for glycine. The amino acid substituted from large-sized arginine (hydrophilic, positively charged) to smaller-sized glycine (hydrophobic, neutral) at position 325 which is close by the main catalytic active sides of OSGEP and predicted to affect its interaction with tRNA.

Supplementary Figure S2 PCA plot of the two first principal components.

Supplementary Figure S3 Expression profiles.

Supplementary Figure S4 Top canonical pathways.

Supplementary Figure S5 Comparative depiction of location and function.

Supplementary Figure S6 Top pathways of genes of the downregulated proteins.

Supplementary Figure S7 Top pathways of genes of the upregulated proteins.
