# Supplementary material for: Genomic, Proteomic, and Phenotypic Spectrum of Novel O-Sialoglycoprotein Endopeptidase Variant in Four Affected Individuals With Galloway-Mowat Syndrome
Source: Front Genet. 2022 Jun 23;13:806190. doi: 10.3389/fgene.2022.806190 (PMC9259880; doi:10.3389/fgene.2022.806190)
Supplement: Supplementary file 1 [file DataSheet1.pdf]

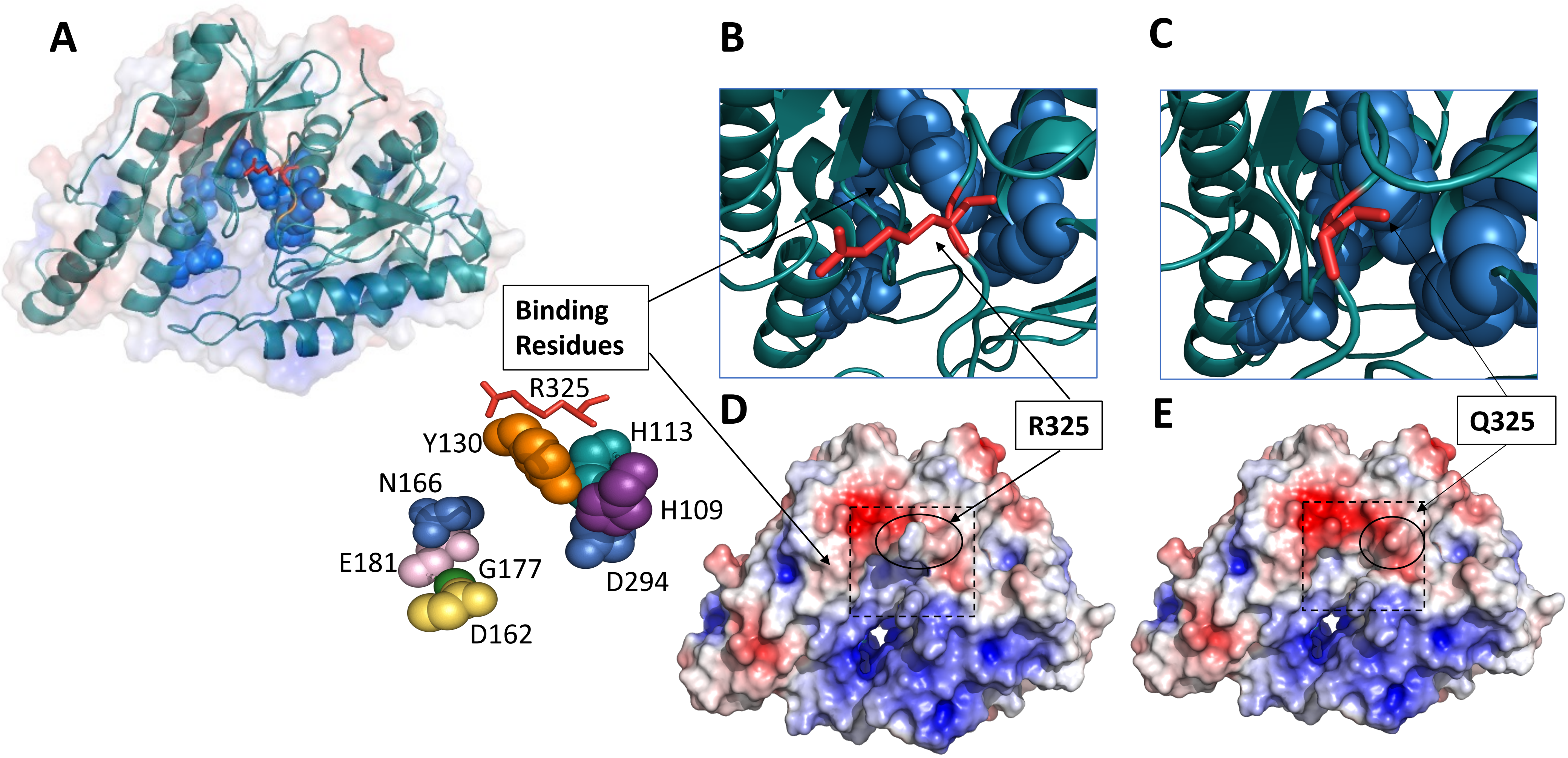

Figure S1: OSGEP Protein 3D Model.

Figure S2: PCA plot of the two first principal components. Both together explained 84.2% of the selected spot's variability. Colored dots and numbers are the representation of gels and spots, respectively.

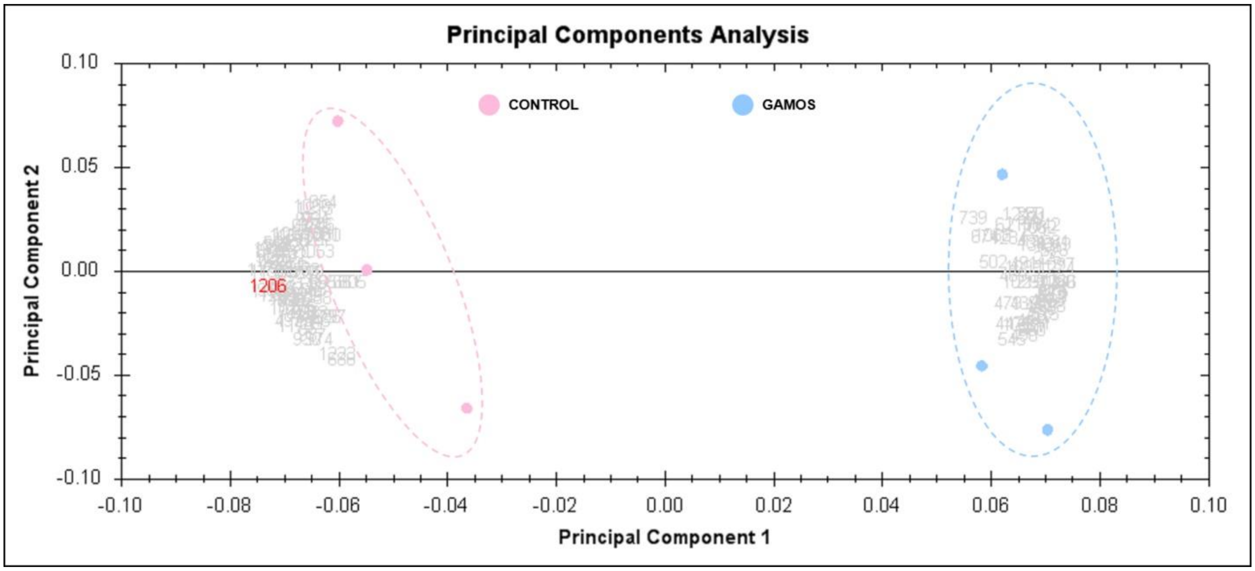

Figure S3: Expression profiles, separated into clusters of expression patterns, indicating the number of spots for each cluster. Each line represents the standardized abundance of a spot across all gels and belongs to one of the clusters generated by hierarchical cluster analysis. The spots with increased abundance indicate the 40 proteins up-regulated in GAMOS patient (Figure 7 B). The spots with decreased abundance indicate the 67 proteins down regulated in GAMOS patient (Figure 7 A) (Progenesis Same Spots).

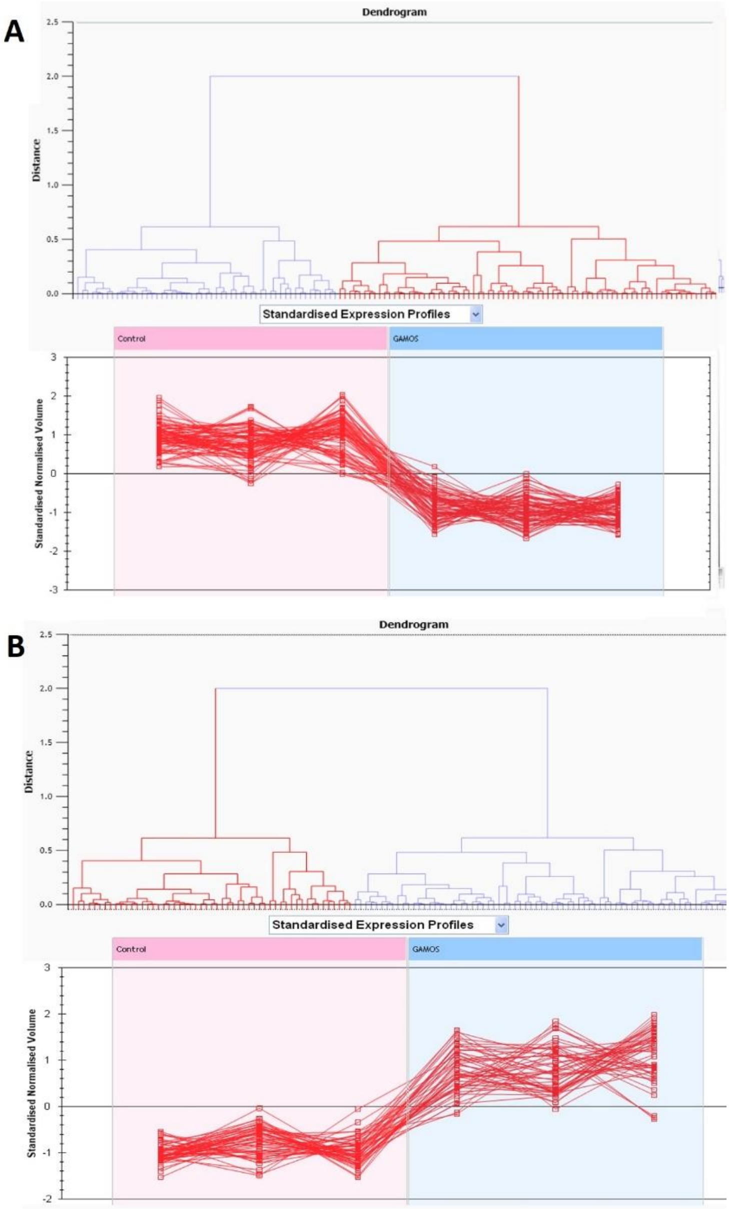

Figure S4: Top Canonical Pathways:

Top Canonical Pathways

Name	p-value	Overlap
RhoA Signaling	6.57E-05	4.1 % 5/123
Remodeling of Epithelial Adherens Junctions	8.83E-05	5.9 % 4/68
Death Receptor Signaling	2.73E-04	4.4 % 4/91
Regulation of Actin-based Motility by Rho	3.09E-04	4.3 % 4/94
Nitric Oxide Signaling in the Cardiovascular System	3.77E-04	4.0 % 4/99

Top Networks

ID	Associated Network Functions	Score
1	Cellular Assembly and Organization, Cellular Function and Maintenance, Tissue Development	53
2	Cancer, Gastrointestinal Disease, Hepatic System Disease	33
3	Cancer, Gastrointestinal Disease, Hepatic System Disease	26
4	Cancer, Neurological Disease, Organismal Injury and Abnormalities	18

Figure S5: A comparative depiction (%) of identified proteins categorized into groups according to their biological Function **A**, Location **B** generated using PANTHER (protein analysis through evolutionary relationships) classification system (http://www.pantherdb.org/)
‘

A. FUNCTION

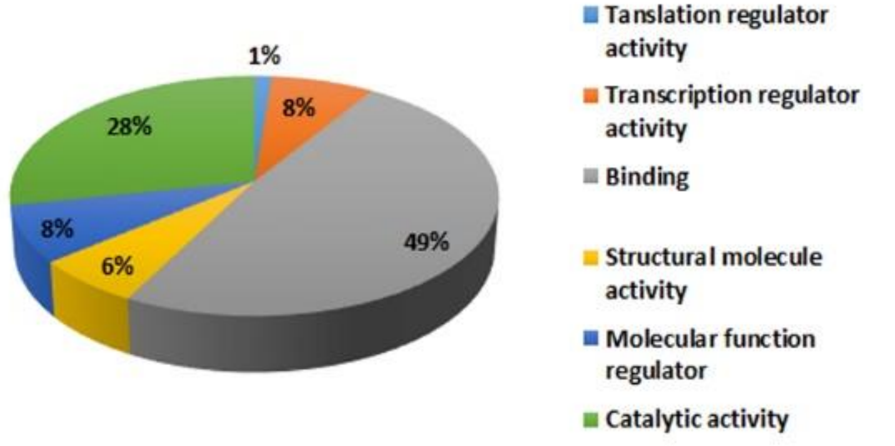

B. LOCATION

‘

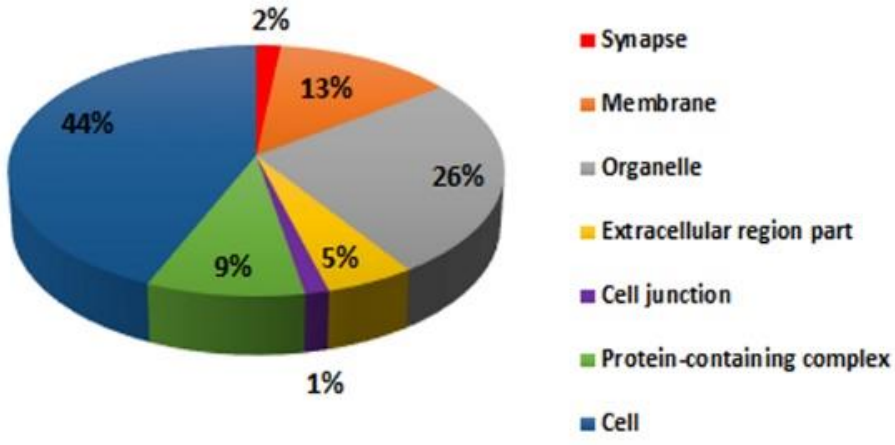

Figure S6: Top pathways of genes of the downregulated proteins.

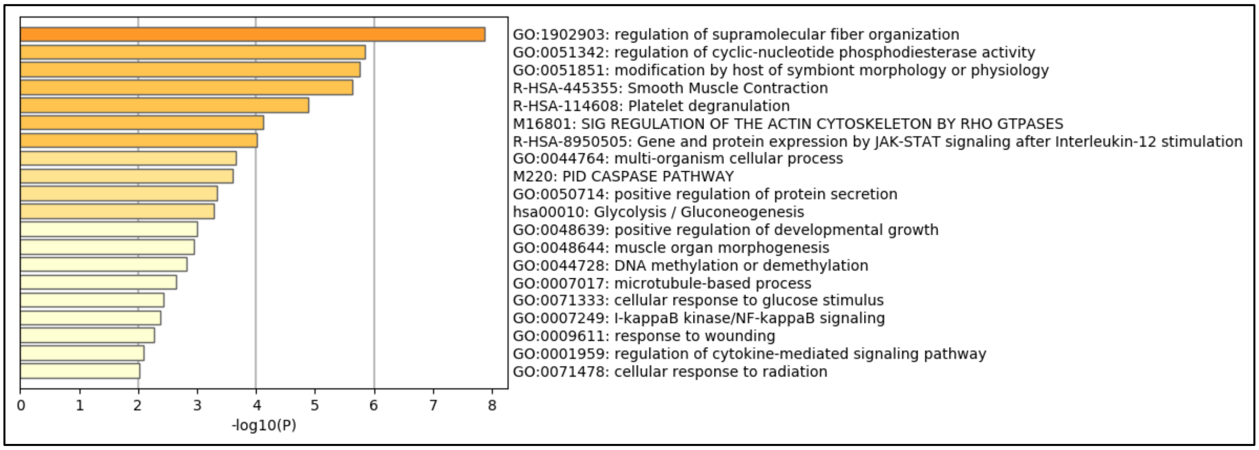

Figure S7: Top pathways of genes of the upregulated proteins.

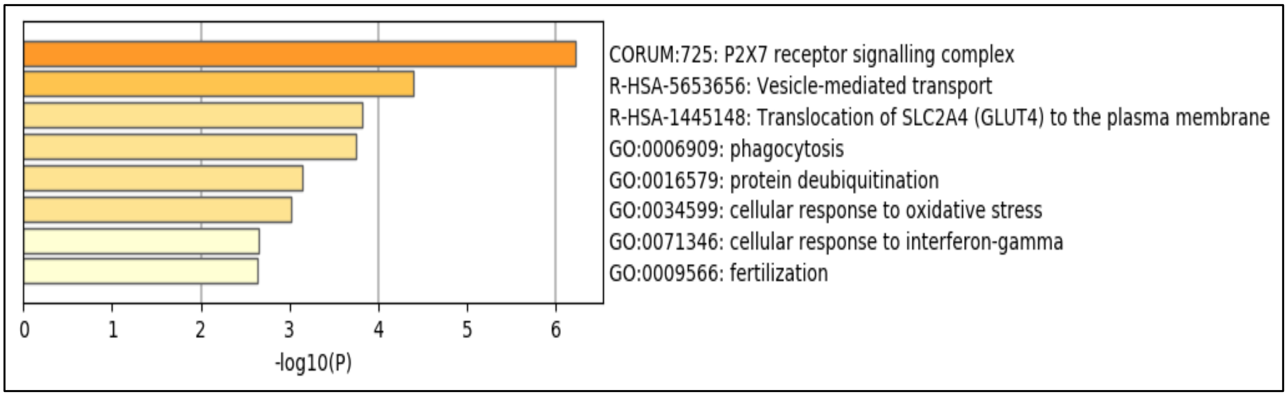

Figure S7: Protein Sequence Alignment.

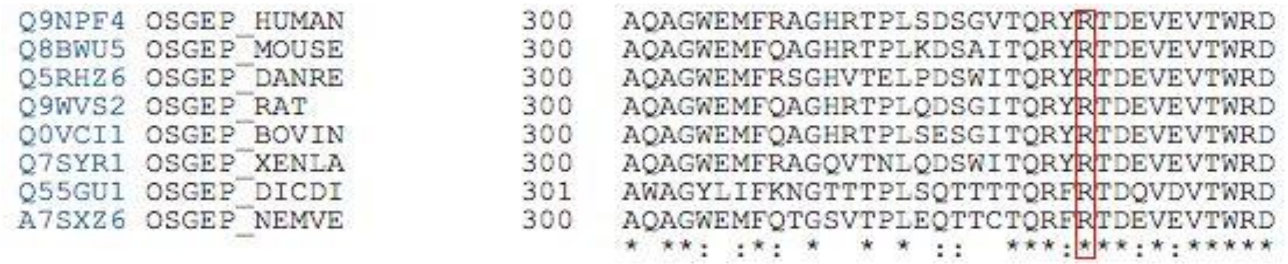

Table S1: Experimental design: 6 samples run on 3-2D PAGE gels, samples were labeled randomly with Cy3 and Cy5, and a pooled sample was used as an internal standard and was stained with Cy2 (C: Control samples, G: GAMOS patient G1-G3 triplicates).

Gel number	Cy3	Cy5	Cy2
G1	C1	G1	Pooled Sample
G2	G2	C2	Pooled Sample
G3	C3	G3	Pooled Sample

Table S2: Mass spectrometry list of significant differentially abundant proteins between control and GAMOS patient identified in samples using 2D-DIGE with. Protein name, accession number, Mascot score, MS % coverage, protein MW and pI values according to Uniprot database are listed.

Sl no:	Spot No[a]	Accession No[b]	Protein Name	MASCOT ID	Pi[a]	MW[b]	Cov[c]%	Score[d]
1	1206	P08779	Keratin, type I cytoskeletal 16	K1C16_HUMAN	4.99	51578	30	64
2	960	O95411	TGFB1-induced anti-apoptotic factor 1	TIAF1_HUMAN	8.35	12691	68	64
3	1019	P78332	RNA-binding protein 6	RBM6_HUMAN	5.93	129129	15	58
4	1173	P49189	4-trimethylaminobutyraldehyde dehydrogenase	AL9A1_HUMAN	5.69	54679	30	57
5	1163	Q2M1K9	Zinc finger protein 423	ZN423_HUMAN	6.43	148787	16	60
6	1138	Q96NU7	Probable imidazolonepropionase	HUTI_HUMAN	6.15	47253	24	58
7	929	Q9GZT9	Egl nine homolog 1	EGLN1_HUMAN	8.83	46847	27	68
8	193	Q15545	Transcription initiation factor TFIID subunit 7	TAF7_HUMAN	5.07	46348	24	57
9	1312	P09382	Galectin-1	LEG1_HUMAN	5.34	15048	82	151
10	1097	P67936	Tropomyosin alpha-4 chain	TPM4_HUMAN	4.67	28619	55	163
11	1200	Q96P63	Serpin B12	SPB12_HUMAN	5.36	46646	37	88
12	225	P14625	Endoplasmin	ENPL_HUMAN	4.76	92696	23	139
13	1233	P62937	Peptidyl-prolyl cis-trans isomerase A	PPIA_HUMAN	7.68	18229	85	57
14	759	Q13489	Baculoviral IAP repeat-containing protein 3	BIRC3_HUMAN	5.71	69582	20	
15	512	Q13882	Protein-tyrosine kinase 6	PTK6_HUMAN	6.56	52371	19	66
16	1042	P08758	Annexin A5	ANXA5_HUMAN	4.94	35971	62	191
17	221	Q14247	Src substrate cortactin	SRC8_HUMAN	5.24	61720	18	59
18	1188	O60812	Heterogeneous nuclear ribonucleoprotein C-like 1	HNRC1_HUMAN	4.93	32180	36	57
19	497	Q01804	OTU domain-containing protein 4	OTUD4_HUMAN	6.25	124823	13	58
20	972	P04083	Annexin A1	ANXA1_HUMAN	6.57	38918	67	216
21	1179	Q5SVZ6	Zinc finger MYM-type protein 1	ZMYM1_HUMAN	7.51	131542	15	60
22	801	P60709	Actin, cytoplasmic 1	ACTB_HUMAN	5.29	42052	77	162
23	1082	Q06830	Peroxiredoxin-1	PRDX1_HUMAN	8.27	22324	87	234
24	838	P07355	Annexin A2	ANXA2_HUMAN	7.57	38808	69	299
25	1110	A6NMY6	Putative annexin A2-like protein	AXA2L_HUMAN	6.49	38806	73	339
26	116	Q6XZB0	Lipase member I	LIPI_HUMAN	9.21	53926	24	60
27	812	P04406	Glyceraldehyde-3-phosphate dehydrogenase	G3P_HUMAN	8.57	36201	69	211
28	675	Q6ZSJ8	Uncharacterized protein C1orf122	CA122_HUMAN	6.29	11521	40	59
29	1101	P68104	Elongation factor 1-alpha 1	EF1A1_HUMAN	9.10	50451	34	75
30	559	Q14980	Nuclear mitotic apparatus protein 1	NUMA1_HUMAN	5.63	239199	14	58

31	749	Q9Y6X1	Stress-associated endoplasmic reticulum protein 1	SERP1_HUMAN	8.75	4652	65	131
32	478	Q16587	Zinc finger protein 74	ZNF74_HUMAN	8.90	74115	33	58
33	1092	P23528	Cofilin-1	COF1_HUMAN	8.22	18719	80	110
34	947	Q9BXR6	Complement factor H-related protein 5	FHR5_HUMAN	6.81	66430	23	60
35	677	Q9NWA0	Mediator of RNA polymerase II transcription subunit 9	MED9_HUMAN	6.84	16506	28	58
36	1162	Q9HB19	Pleckstrin homology domain-containing family A member 2	PKHA2_HUMAN	8.92	47852	44	63
37	913	P08865	40S ribosomal protein SA	RSSA_HUMAN	4.79	32947	40	110
38	996	Q9NVM9	Integrator complex subunit 13	INT13_HUMAN	6.24	81144	20	58
39	1133	P0DP23	Calmodulin-1	CALM1_HUMAN	4.09	16827	50	58
40	230	Q9UK80	Ubiquitin carboxyl-terminal hydrolase 21	UBP21_HUMAN	7.71	16598	41	58
41	1207	P23284	Peptidyl-prolyl cis-trans isomerase B	PPIB_HUMAN	9.42	23785	58	130
42	501	Q9Y4A5	Transformation/transcription domain-associated protein	TRRAP_HUMAN	8.49	441766	14	66
43	930	Q8NHY6	Zinc finger protein 28 homolog	ZFP28_HUMAN	9.44	100809	25	58
44	337	P09874	Poly [ADP-ribose] polymerase 1	PARP1_HUMAN	8.99	113811	20	58
45	853	P07355	Annexin A2	ANXA2_HUMAN	7.57	38808	82	239
46	1266	P07737	Profilin-1	PROF1_HUMAN	8.44	15216	69	107
47	1062	Q9H254	Spectrin beta chain, non-erythrocytic 4	SPTN4_HUMAN	5.72	290005	19	63
48	455	O00186	Syntaxin-binding protein 3	STXB3_HUMAN	7.98	68633	27	58
49	599	Q8TD57	Dynein heavy chain 3, axonemal	DYH3_HUMAN	6.04	473776	9	63
50	513	Q9UEW3	Macrophage receptor MARCO	MARCO_HUMAN	8.95	52968	32	66
51	122	Q9Y295	Developmentally-regulated GTP-binding protein 1	DRG1_HUMAN	9.00	40802	31	57
52	1343	Q8NCI6	Beta-galactosidase-1-like protein 3	GLBL3_HUMAN	9.05	75118	14	58
53	1099	P07355	Annexin A2	ANXA2_HUMAN	7.57	38808	43	113
54	993	P47756	F-actin-capping protein subunit beta	CAPZB_HUMAN	5.36	31616	59	168
55	442	Q16587	Zinc finger protein 74	ZNF74_HUMAN	8.90	74115	37	58
56	483	O15144	Actin-related protein 2/3 complex subunit 2	ARPC2_HUMAN	6.84	34426	31	61
57	937	Q9BYG7	Protein maestro	MSTRO_HUMAN	9.93	29149	25	57
58	219	Q330K2	NADH dehydrogenase (ubiquinone) complex I, assembly factor 6	NDUF6_HUMAN (CHO_38)	9.44	38551	30	59
59	714	P14618	Pyruvate kinase PKM	KPYM_HUMAN	7.96	58470	38	98
60	481	Q7Z3Z4	Piwi-like protein 4	PIWL4_HUMAN	9.09	97838	27	58
61	507	Q8WXH0	Nesprin-2	SYNE2_HUMAN	5.26	80187	8	60
62	563	Q8TD57	Dynein heavy chain 3, axonemal	DYH3_HUMAN	6.04	473776	14	60
63	374	Q3ZCT8	Kelch repeat and BTB domain-containing protein 12	KBTBC_HUMAN	5.65	72077	25	58
64	1023	Q15022	Polycomb protein SUZ12	SUZ12_HUMAN	8.98	83744	20	60
65	500	P52790	Hexokinase-3	HXK3_HUMAN	5.23	100616	19	58
66	491	Q02556	Interferon regulatory factor 8	IRF8_HUMAN	6.38	49123	44	58
67	971	Q9UEW3	Macrophage receptor MARCO	MARCO_HUMAN	8.95	52968	23	60
68	1246	P22392	Nucleoside diphosphate kinase B	NDKB_HUMAN	8.52	17401	87	136
69	346	Q86UR5	Regulating synaptic membrane exocytosis protein 1	RIMS1_HUMAN	9.68	190154	15	58

70	445	Q7Z6M3	Allergin-1	MILR1_HUMAN	7.92	39395	41	59
71	543	Q9NV70	Exocyst complex component 1	EXOC1_HUMAN	6.17	102772	23	58
72	555	Q9UKT9	Zinc finger protein Aiolos	IKZF3_HUMAN	6.11	59012	30	59
73	439	P09874	Poly [ADP-ribose] polymerase 1	PARP1_HUMAN	8.99	113811	22	7
74	964	P09651	Heterogeneous nuclear ribonucleoprotein A1	ROA1_HUMAN	9.17	38837	51	142
75	490	P52790	Hexokinase-3	HXK3_HUMAN	5.23	100616	11	57
76	1055	Q4G0A6	Probable ubiquitin carboxyl-terminal hydrolase MINDY-4	MINY4_HUMAN	6.47	85261	18	65
77	661	Q9NS84	Carbohydrate sulfotransferase 7	CHST7_HUMAN	9.72	54803	23	57
78	896	P63244	Receptor of activated protein C kinase 1	RACK1_HUMAN	7.60	35511	63	141
79	739	P07437	Tubulin beta chain	TBB5_HUMAN	4.78	50095	52	152
80	1106	Q01995	Transgelin	TAGL_HUMAN	8.87	22653	51	66
81	1096	P60709	Actin, cytoplasmic 1	ACTB_HUMAN	5.29	42052	55	92
82	805	P63261	Actin, cytoplasmic 2	ACTG_HUMAN	5.31	42108	84	283
83	1213	P60981	Destrin	DEST_HUMAN	8.06	18950	53	58
84	545	Q8NDG6	ATP-dependent RNA helicase TDRD9	TDRD9_HUMAN	6.62	157124	17	58
85	865	P04406	Glyceraldehyde-3-phosphate dehydrogenase	G3P_HUMAN	8.57	36201	63	206
86	222	P08238	Heat shock protein HSP 90-beta	HS90B_HUMAN	4.97	83554	54	170
87	410	Q14980	Nuclear mitotic apparatus protein 1	NUMA1_HUMAN	5.63	239199	18	59
88	813	Q13796	Protein Shroom2	SHRM2_HUMAN	6.64	177500	18	59
89	738	O43852	Calumenin	CALU_HUMAN	4.47	37198	25	70
90	554	P49767	Vascular endothelial growth factor C	VEGFC_HUMAN	7.77	49019	32	58
91	1059	P63104	14-3-3 protein zeta/delta	1433Z_HUMAN	4.73	27899	65	184
92	576	Q96NN9	Apoptosis-inducing factor 3	AIFM3_HUMAN	9.20	67376	23	60
93	1280	Q9Y2L8	Zinc finger protein with KRAB and SCAN domains 5	ZKSC5_HUMAN	7.43	98994	19	69
94	1281	Q8N806	Putative E3 ubiquitin-protein ligase UBR7	UBR7_HUMAN	4.7	49336	13	58
95	943	P07951	Tropomyosin beta chain	TPM2_HUMAN	4.66	32945	53	142
96	506	P50990	T-complex protein 1 subunit theta	TCPQ_HUMAN	5.42	60153	41	59
97	642	Q6P5S0	C18orf34 protein	Q6P5S0_HUMAN	6.34	102575	18	50
98	499	Q15233	Non-POU domain-containing octamer-binding protein	NONO_HUMAN	9.01	54311	25	58
99	1027	Q86UB2	Basic immunoglobulin-like variable motif-containing protein	BIVM_HUMAN	9.15	57479	18	57
100	1308	Q16891	MICOS complex subunit MIC60	MIC60_HUMAN	6.08	84025	24	58
101	988	Q96LR7	Uncharacterized protein C2orf50	CB050_HUMAN	9.51	17941	34	59
102	132	P03952	Plasma kallikrein	KLKB1_HUMAN	8.60	73433	28	61
103	511	Q9BX26	Synaptonemal complex protein 2	SYCP2_HUMAN	9.01	177239	13	59
104	974	P29692	Elongation factor 1-delta	EF1D_HUMAN	4.9	31217	60	130
105	621	Q8WVF1	Protein OSCP1	OSCP1_HUMAN	5.75	43300	34	58
106	1060	P09493	Tropomyosin alpha-1 chain	TPM1_HUMAN	4.69	32746	42	95
107	447	P11142	Heat shock cognate 71 kDa protein	HSP7C_HUMAN	5.37	71082	45	98

[a] Protein accession number for SWISSPROT Database.

[a] Theoretical isoelectric point.

[b] Theoretical relative mass.

[c] MASCOT coverage
[d] MASCOT score
